# Stat3 and c-Myc Genome-Wide Promoter Occupancy in Embryonic Stem Cells

**DOI:** 10.1371/journal.pone.0003932

**Published:** 2008-12-11

**Authors:** Benjamin L. Kidder, Jim Yang, Stephen Palmer

**Affiliations:** 1 EMD Serono Research Institute, Inc., Rockland, Massachusetts, United States of America; 2 EMD Serono Research Center, Inc., Billerica, Massachusetts, United States of America; City of Hope Medical Center, United States of America

## Abstract

Embryonic stem (ES) cell pluripotency is regulated in part by transcription factor (TF) pathways that maintain self-renewal and inhibit differentiation. Stat3 and c-Myc TFs are essential for maintaining mouse ES cell self-renewal. c-Myc, together with Oct4, Sox2, and Klf4, is a reprogramming factor. While previous studies have investigated core transcriptional circuitry in ES cells, other TF pathways that promote ES cell pluripotency have yet to be investigated. Therefore, to further understand ES cell transcriptional networks, we used genome-wide chromatin immunoprecipitation and microarray analysis (ChIP-chip) to map Stat3 and c-Myc binding targets in ES cells. Our results show that Stat3 and c-Myc occupy a significant number of genes whose expression is highly enriched in ES cells. By comparing Stat3 and c-Myc target genes with gene expression data from undifferentiated ES cells and embryoid bodies (EBs), we found that Stat3 binds active and inactive genes in ES cells, while c-Myc binds predominantly active genes. Moreover, the transcriptional states of Stat3 and c-Myc targets are correlated with co-occupancy of pluripotency-related TFs, polycomb group proteins, and active and repressive histone modifications. We also provide evidence that Stat3 targets are differentially expressed in ES cells following removal of LIF, where culture of ES cells in the absence of LIF resulted in downregulation of Stat3 target genes enriched in ES cells, and upregulation of lineage specific Stat3 target genes. Altogether, we reveal transcriptional targets of two key pluripotency-related genes in ES cells – Stat3 and c-Myc, thus providing further insight into the ES cell transcriptional network.

## Introduction

Pluripotent embryonic stem (ES) cells, derived from mammalian preimplantation embryos, can be cultured indefinitely *in vitro* and have the ability to differentiate into all somatic and germ cell lineages [1], [2]. ES cell pluripotency can be maintained in the presence of extrinsic signals such as leukemia inhibitory factor (LIF) in addition to fetal calf serum (FCS). LIF belongs to the interleukin-6 family of cytokines that signals through heterodimerization of receptors gp130 and LIF-R, resulting in activation of Jak, and phosphorylation of gp130 and LIF-R [3]. Subsequent Stat3 phosphorylation results in nuclear translocation and target gene transcriptional activation or repression [4]. Stat3 is necessary to maintain ES cells in a self-renewing state [5]–[7]. Expression of Stat3 maintains self-renewal in the absence of LIF [7], while disruption of Stat3 results in ES cell differentiation [5]. While these studies demonstrate that Stat3 is essential for ES cell pluripotency, downstream targets of LIF/Jak/Stat3 signaling have not been fully identified.

Although LIF-signaling is sufficient to maintain ES cell pluripotency in serum conditions, LIF is insufficient to maintain ES cell pluripotency in serum-free conditions. Additional factors present in serum contribute to maintenance of pluripotency. Recently it was shown that a combination of BMP4 and LIF has the ability to maintain ES cell self-renewal in serum-free conditions [8]. BMP4/Smad signaling has been shown to drive expression of inhibitor of differentiation (Id) genes, and forced expression of Id genes in ES cells is sufficient for maintaining self-renewal in the absence of BMP4 [8]. Moreover, canonical Wnt signaling has been shown to promote ES cell self-renewal in a LIF- and serum-independent mechanism, where GSK3 inhibition activates β-catenin resulting in nuclear translocation and association with TCF/LEF transcription factors [9]. Therefore, BMP and Wnt signaling are important in maintaining ES cell pluripotency. c-Myc is one target of Wnt signaling, in which c-Myc is activated in response to β-catenin/TCF/LEF transcription factor activity [10]. c-Myc has been shown to maintain ES cell pluripotency in the absence of LIF [11]. It has also been suggested that Stat3 and c-Myc share similar downstream effector genes [11]. Moreover, pluripotency can be conferred upon mouse and human somatic cells through overexpression of c-Myc and three other transcription factors (Oct4, Sox2, and Klf4) [12]–[15]. Evaluating transcription factor pathways involved in acquiring and maintaining a pluripotent state is critical in understanding mechanisms that contribute to pluripotency, acquiring a pluripotent state, and lineage specific differentiation. Thus, identification of Stat3 and c-Myc targets will lend further insight into transcriptional networks that govern ES cells pluripotency.

Recently, numerous genome-wide studies have evaluated promoter binding of core pluripotency genes Oct4, Sox2, and Nanog [16], [17], in addition to transcription factors such as Klf proteins [18], Tcf3 [19], and other ES cell enriched genes [20], [21]. Results from these genome-wide chromatin immunoprecipitation and microarray analysis (ChIP-chip) and sequencing (ChIP-seq) experiments have been useful in constructing a framework of the ES cell transcriptional network. Continued efforts to build upon knowledge of ES cell transcriptional networks will strengthen our understanding of ES cell pluripotency. To this end we evaluated Stat3 and c-Myc target genes in ES cells using genome-wide ChIP-chip analysis.

We report that Stat3 and c-Myc occupy a significant number of genes in ES cells that function in biological processes such as gene expression and development. Interestingly, Stat3 and c-Myc bind many ES cell enriched genes. By comparing Stat3 and c-Myc target genes with gene expression data from undifferentiated ES cells and differentiated embryoid bodies (EBs), we found that Stat3 binds both active and inactive genes in ES cells, while c-Myc binds predominantly active genes. Moreover, Stat3 and c-Myc co-occupancy with pluripotency-related genes and regions of active histone modifications is correlated with target gene activity, while Stat3 co-occupancy with polycomb repressive complex proteins (PcG), or regions with repressive bivalent histone modifications (H3K4/27me3) is correlated with target gene inactivity in ES cells. Our results also reveal that Stat3 targets are differentially expressed in ES cells in the absence of LIF. These results demonstrate an important role for Stat3 and c-Myc in regulating pluripotency-related gene expression in ES cells.

## Results

### Identification of Stat3 and c-Myc promoter binding in ES cells

**Figure 1:**
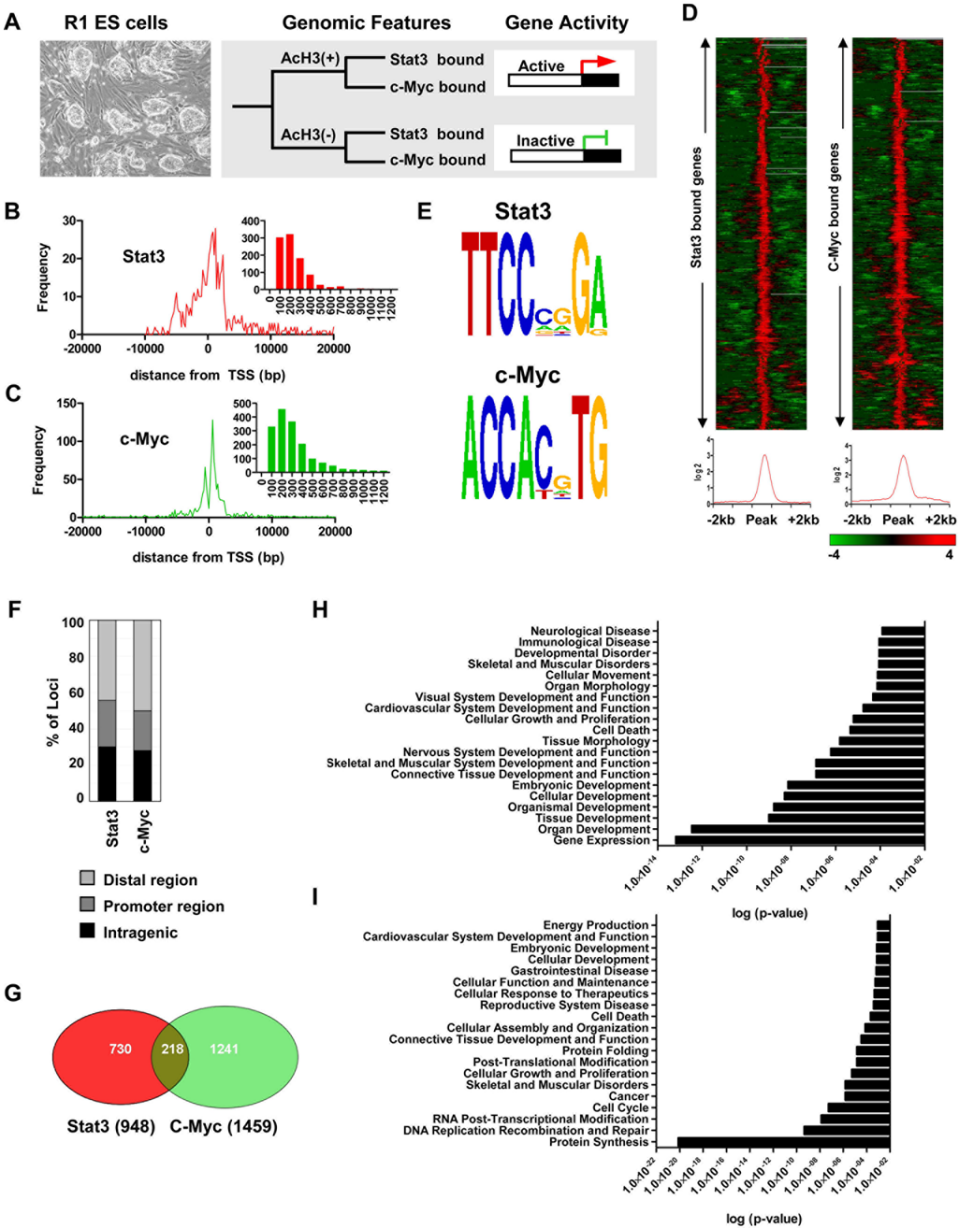
Genome-wide analysis of Stat3 and c-Myc binding in ES cells. (A) Experimental design. See methods and text for details. ChIP-chip analysis was used to map Stat3 and c-Myc genomic binding and regions of AcH3. Genes with AcH3 marks were considered actively transcribed. Distribution of (B) Stat3 and (C) c-Myc binding regions relative to the nearest transcription start site (TSS) of known genes. Inset graph shows length of (B) Stat3 and (C) c-Myc enriched DNA sequences. Y-axis represents frequency of binding and X-axis represents length of binding (bp). (D) Clustering performed on Stat3 and c-Myc binding profiles centered on enrichment peaks [−2kb, +2kb]. Average binding profiles are shown below the heat map. (E) Stat3 and c-Myc enriched motifs identified using the de novo motif discovery algorithm Weeder. (F) Stat3 and c-Myc binding distribution relative to genic and non-genic regions. (G) Venn diagram showing overlap of Stat3 and c-Myc bound genes. Gene ontology (GO) functional annotation of (H) Stat3 and (I) bound genes was performed using Ingenuity Pathway Analysis (IPA). The 20 most significant biological process GO terms are shown.

To identify genome-wide promoter-binding regions of Stat3 and c-Myc in ES cells, we used chromatin immunoprecipitation and DNA microarray analysis (ChIP-chip). Stat3 is a downstream component of leukemia inhibitory factor (LIF) signaling, which is integral in maintaining ES cells in an undifferentiated state. c-Myc promotes ES cell self-renewal in the absence of LIF [11] and is also a reprogramming factor [12], [15]. By mapping genomic regions of Stat3 and c-Myc promoter binding we aim to evaluate the binding patterns of these TFs and how they are connected in the ES cell transcriptional network. ChIP-chip analysis of histone H3 acetylation was used as a marker for actively transcribed DNA sequences. Chromatin immunoprecipitation was performed using antibodies specific to the TFs Stat3 and c-Myc, and AcH3 (Figure 1A, see methods). ChIP-enriched DNA sequences were amplified and hybridized to high-density DNA promoter tiling arrays spanning regions of 28,000 murine promoter regions (see methods). Regions of Stat3 and c-Myc genomic binding were determined using Tilemap [22] and annotated to the nearest transcriptional start site (TSS) (Table S1 and S2). We found 948 genes bound by Stat3 and 1459 genes bound by c-Myc. Genomic regions of Stat3 and c-Myc binding are located near TSSs with many located within 5kb of TSSs (Stat3 - 54%; c-Myc - 60%) (Figure 1B–C). Stat3 has an average length of binding of 239bp while c-Myc has an average length of binding of 339bp (Figure 1B–C). Stat3 and c-Myc binding profiles are shown in Figure 1D. To evaluate the consensus-binding motifs of Stat3 and c-Myc TFs we used the Weeder algorithm [23]. Sequences from 100 binding peaks with the greatest number of good probes were chosen from our Stat3 and c-Myc dataset and imported into Weeder. We obtained the known consensus binding motifs of Stat3 (5′-TTCCCGGA-3′) and c-Myc (5′-CACGTG-3′) from our Stat3 and c-Myc dataset (Figure 1E). These consensus binding motifs are similar to Stat3 and c-Myc binding sequences reported previously [20], [21], [24], [25].

Relative to c-Myc, Stat3 binds a greater percentage of promoter and intragenic regions, and a smaller percentage of distal regions (Figure 1F). Co-occupancy of Stat3 and c-Myc target genes was found at 218 promoters (Figure 1G) including genes enriched in ES cells. Stat3 and c-Myc target genes were functionally annotated using Ingenuity Pathway Analysis (IPA). Biological processes enriched in Stat3 targets include gene expression, organ-, tissue-, organismal-, and embryonic-development, and cellular growth and proliferation (Figure 1H). Biological processes enriched in c-Myc targets include protein synthesis, cell cycle, cancer, cellular development, and embryonic development (Figure 1I).

### Stat3 and c-Myc bind ES cell enriched genes

**Figure 2:**
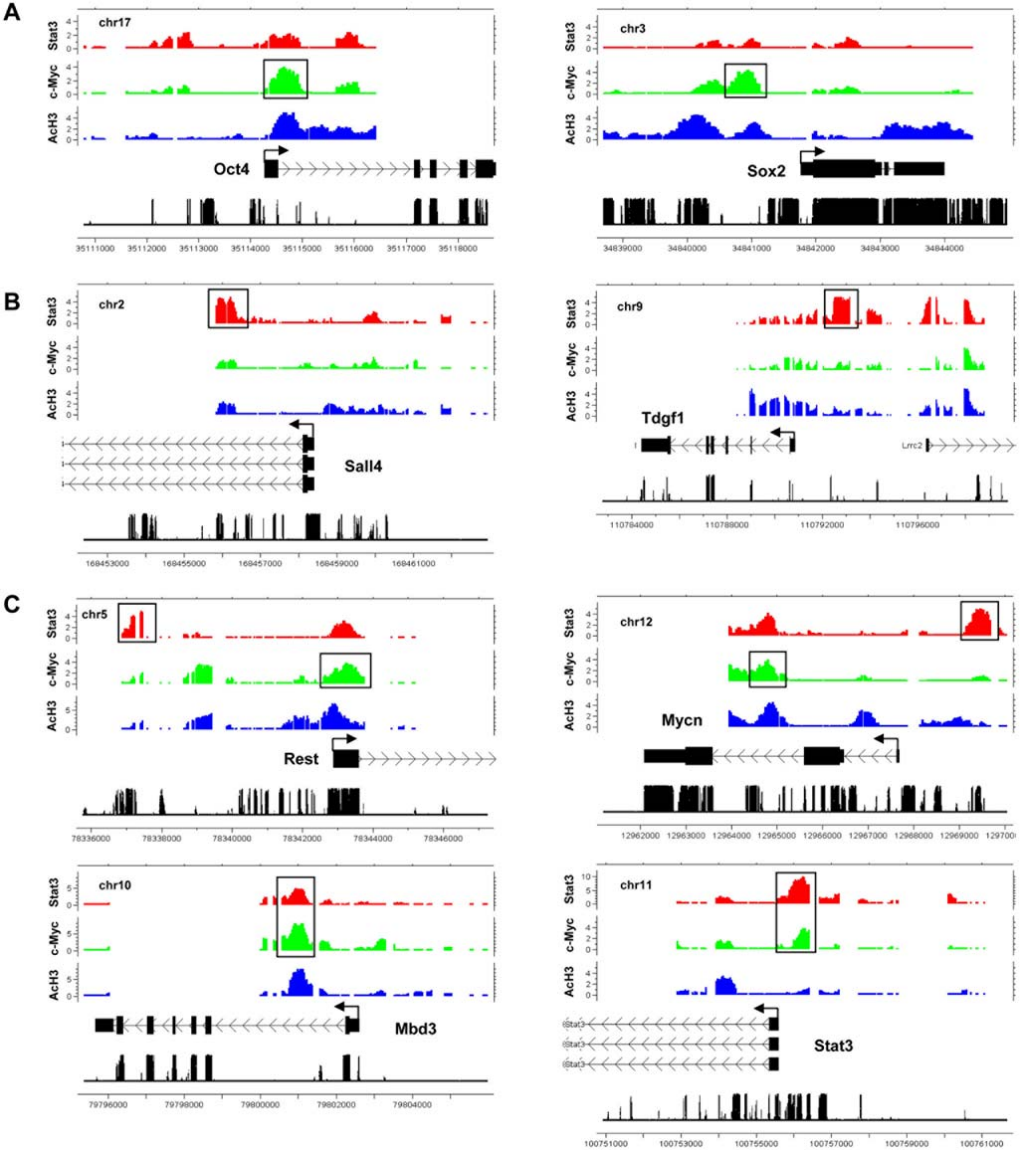
Genomic maps of Stat3 and c-Myc binding at pluripotency-related gene loci. Stat3 and c-Myc genomic binding site and acetylated histone H3 (AcH3) profiles at ES cell enriched gene loci. Representative (A) c-Myc bound genes (Oct4, Sox2), (B) Stat3 bound genes (Sall4, Tdgf1), and Stat3 and c-Myc co-bound genes (Rest, Mycn, Mbd3, Stat3). Moving average (MA) enrichment values adjusted to log2 are shown. Chromosome number, sequence position, and conservation are also indicated on the plot.

**Figure 3:**
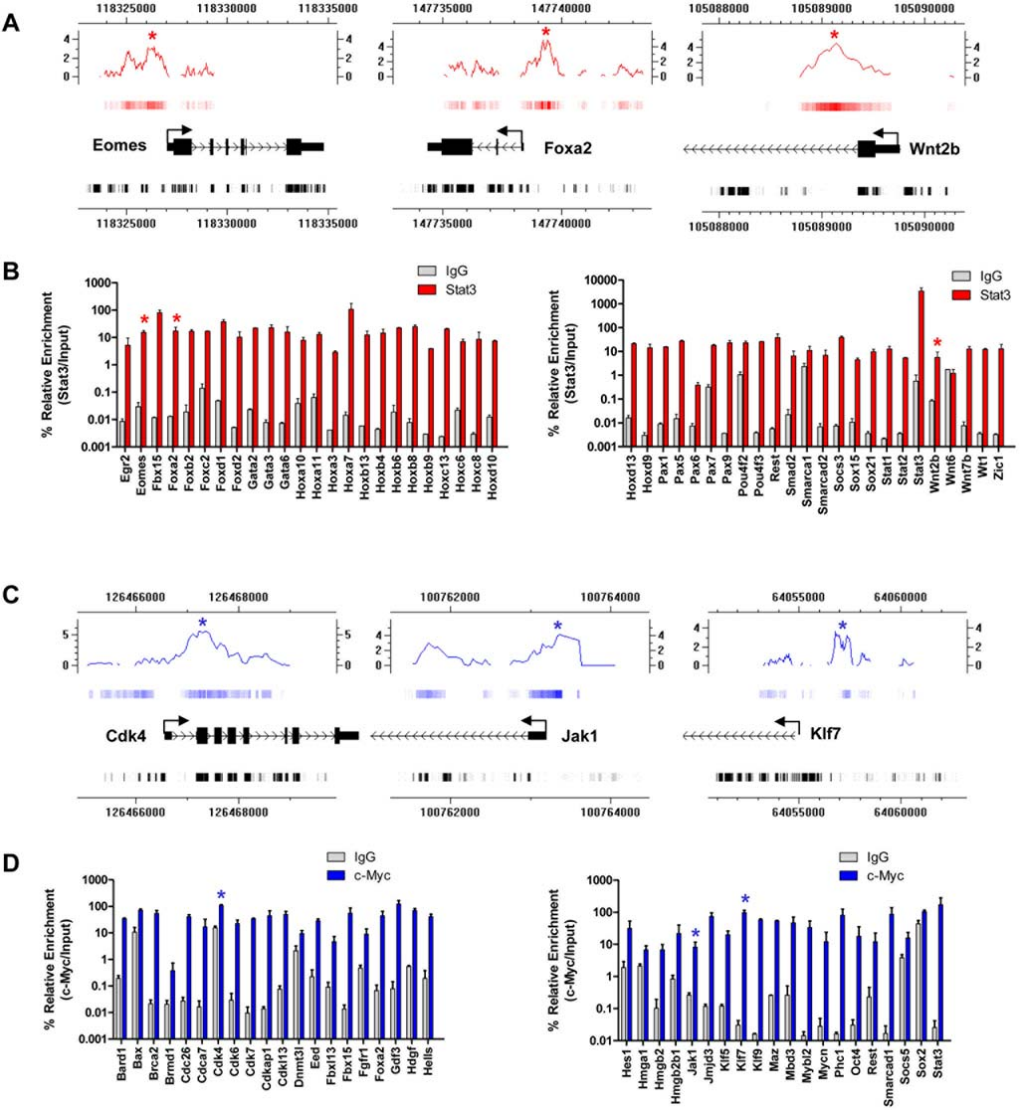
Q-PCR analysis of Stat3 and c-Myc ChIP-enriched regions. (A) Representative Stat3 genomic binding sites confirmed by Q-PCR. Red stars denote ChIP-chip enriched DNA sequences that were amplified using Q-PCR. (B) Confirmation of Stat3-ChIP enriched TF binding sites by Q-PCR. (C) Representative c-Myc genomic binding sites confirmed by Q-PCR. Blue stars denote ChIP-chip enriched DNA sequences that were amplified using Q-PCR. (D) Confirmation of c-Myc-ChIP enriched TF binding sites by Q-PCR. Q-PCR was performed on non-amplified Stat3-, c-Myc-, and IgG-ChIP enriched and Input DNA. Results are shown as percent enrichment relative to Input. MA enrichment values adjusted to log2 are shown in addition to sequence position. Stat3 and c-Myc enrichment are shown as red and blue heat maps respectively, while conservation is shown as a grayscale heat map.

Genome-wide analysis of promoter binding revealed that Stat3 and c-Myc occupy many pluripotency-related genes and genes highly enriched in ES cells. Genes bound by c-Myc include Oct4, Sox2, Mycn, Rest, Stat3, Mbd3, Jmjd3, Gdf3, Fbxo15, Klf5, Klf7, and Klf9 (Figure 2A, Table S2). Genes bound by Stat3 include Sall4, Mycn, Rest, Stat3, Mbd3, Jmjd3, Tdgf1, and Fbxo15 (Figure 2B, Table S1). Genes co-occupied by Stat3 and c-Myc also expressed highly in ES cells include Mycn, Rest, Stat3, Mbd3, and Jmjd3 (Figure 2C, Table S1 and S2). c-Myc also binds epigenetic regulators such as Dnmt1 and Dnmt3l (Table S2). Stat3 and c-Myc genomic targets were confirmed using Q-PCR on ChIP-enriched DNA sequences (Figure 3). Stat3 and c-Myc ChIP-enriched DNA fragments were consistently over-represented relative to non-immune control ChIP DNA fragments (Figure 3). More than 87% of Stat3 targets were enriched over 100-fold relative to control, while 65% of c-Myc targets were enriched over 100-fold, and 20% were enriched over 10-fold relative to control (Figure 3B,D). These data suggest that Stat3 and c-Myc occupy many ES cell enriched genes.

### Stat3 and c-Myc occupy active and inactive genes in ES cells

Regulation of mammalian gene expression is facilitated in part by posttranslational modifications of histones that contribute to chromatin organization [26]. Acetylation of Histone H3 (AcH3) is associated with actively transcribed euchromatic chromatin regions [26]. To evaluate chromatin regions bound by Stat3 or c-Myc containing histone H3 aceylation we performed ChIP-chip analysis in ES cells using an antibody specific to AcH3 (see methods). AcH3 DNA sequences were found near TSSs of genes highly expressed in ES cells such as Oct4, Nanog, Sox2, Rest, Rex1, Tdgf1, Mycn, Dnmt3l, Dppa2, and Gdf3 (Figure 2 and data not shown). Alternatively, AcH3 was not associated with lineage specific genes repressed in ES cells such as endodermal genes (Gata4, Gata6, Sox11, and Sox17), mesodermal genes (Fabp4, Hand1, Hand2, and Snail), ectodermal genes (Nes, Olig1, Otx2, and Pax5), and trophectodermal genes (Cdx2 and Tead4).

To identify Stat3 and c-Myc bound genes that are actively transcribed, we compared Stat3 and c-Myc target genes with regions of histone H3 acetylation (AcH3). We observed regions of AcH3 and Stat3 or c-Myc binding at many ES cell enriched genes including Oct4, Sox2, Mycn, Rest, Mbd3, and Tdgf1 (Figure 2). While AcH3 is associated with actively transcribed genes, to fully understand target gene activity or inactivity it is necessary to directly evaluate the expression of Stat3 and c-Myc targets in ES cells.

**Figure 4:**
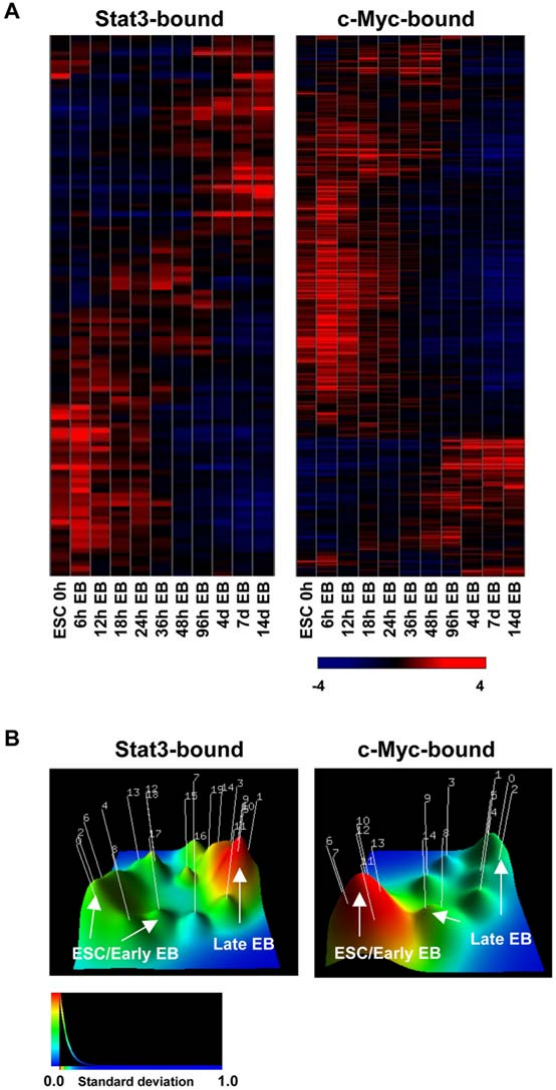
Expression analysis of Stat3 and c-Myc bound genes in ES cells and EBs. (A) Expression analysis of Stat3 and c-Myc target genes in ES cells and differentiated EBs through 14 days. Differentially expressed genes (FDR less than 5%) were compared with Stat3 and c-Myc target genes. (B) Stat3 targets are both active and inactive in ES cells while c-Myc targets are mostly active in ES cells. Three dimensional terrain map of Stat3 and c-Myc target gene expression in ES cells and EBs generated using gCLUTO [28]. Stat3 bound targets are expressed in both undifferentiated ES cells and EB, while more c-Myc targets are expressed higher in undifferentiated ES cells relative to EBs. Peak height is proportional to cluster internal similarity and peak volume is proportional to the number of features within the cluster.

To evaluate the expression profile of Stat3 and c-Myc target genes in ES cells we compared Stat3 and c-Myc target genes with transcriptome data from R1 ES cells and embryoid-body (EB) differentiated ES cells [27]. Hierarchical clustering analysis was performed on differentially expressed genes (p-value less than 5%) also bound by Stat3 or c-Myc (Figure 4A). We found about fifty-percent of Stat3 target genes expressed at elevated levels in undifferentiated ES cells compared with EBs (Figure 4A). On the contrary, the majority of c-Myc target genes exhibited elevated expression in undifferentiated ES cells compared with EBs (Figure 4A). To further investigate the relationship between Stat3 and c-Myc binding and RNA expression in ES cells and EBs we generated a terrain map of Stat3 and c-Myc target gene expression values using gCLUTO (Figure 4B) [28]. Stat3 and c-Myc expression value matrices were clustered into 20 and 10 clusters respectively to best represent the data set. Multidimensional scaling (MDS) identified two main peaks within the Stat3 data set. Stat3 bound genes that were differentially expressed between ES cells and differentiated EBs were clustered into two main groups of similar size, including (1) ES cell and early EB and (2) late EB expression, demonstrating a relatively equal distribution of Stat3 target gene expression between ES cells and EBs (Figure 4B). Conversely, MDS identified one main peak within the c-Myc data set. c-Myc targets were clustered into one main group, including gene expression in ES cells and early EBs (Figure 4B). These results suggest that Stat3 is associated with genes highly expressed in undifferentiated and differentiated ES cells, while c-Myc is predominantly associated with genes highly expressed in undifferentiated ES cells.

**Figure 5:**
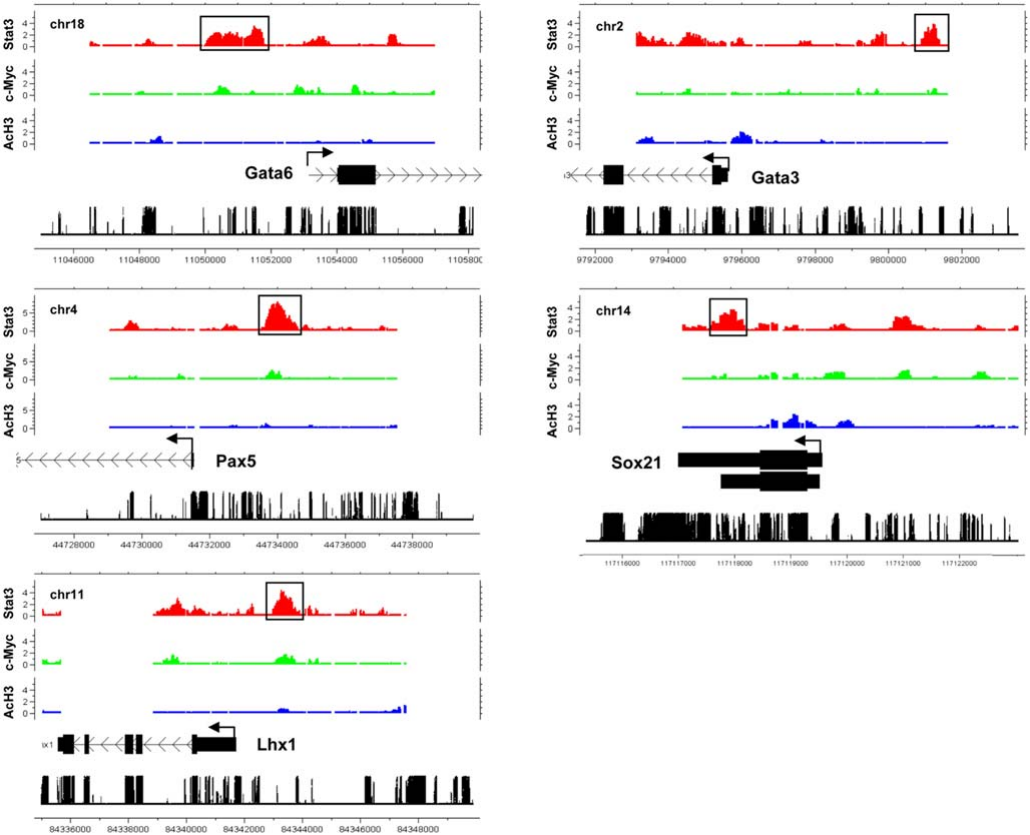
Stat3 binds developmentally repressed genes in ES cells. Genome-wide analysis of Stat3 and c-Myc TF binding sites and histone H3 acetylation (AcH3) at developmentally repressed genes (Gata6, Gata3, Pax5, Sox21, and Lhx1). Moving average (MA) enrichment values adjusted to log2 are shown. Chromosome number, sequence position, and conservation are also indicated on the plot.

About half of Stat3 target genes were expressed at elevated levels in EBs relative to ES cells, with many of these genes lacking significant c-Myc binding or AcH3 histone marks. Stat3 target genes not bound by c-Myc and without AcH3 histone marks include genes expressed in differentiated cells such as Gata2, Gata3, Gata6, Lhx1, Pax5, Sox15, Sox21, and Wnt2b (Figure 5) (Table S1). Stat3 also bound other genes inactive in ES cells including many Hox genes such a Hoxa3, Hoxa10, Hoxa11, Hoxb4, Hoxb6, Hoxb8, hoxb9, Hoxb13, Hoxc6, Hoxc8, Hoxc12, Hoxc13, Hoxd9, Hoxd10, and Hoxd13 (Table S1). In addition to binding Hox genes, Stat3 bound over 50 genes encoding transcriptional regulators with homeodomain motifs. Many of these genes are inactivate in ES cells, suggesting that Stat3 binding represses transcription of developmentally regulated genes in ES cells.

### Gene regulation of Stat3 and c-Myc target genes by co-occupancy of transcription factors and histone modifications

**Figure 6:**
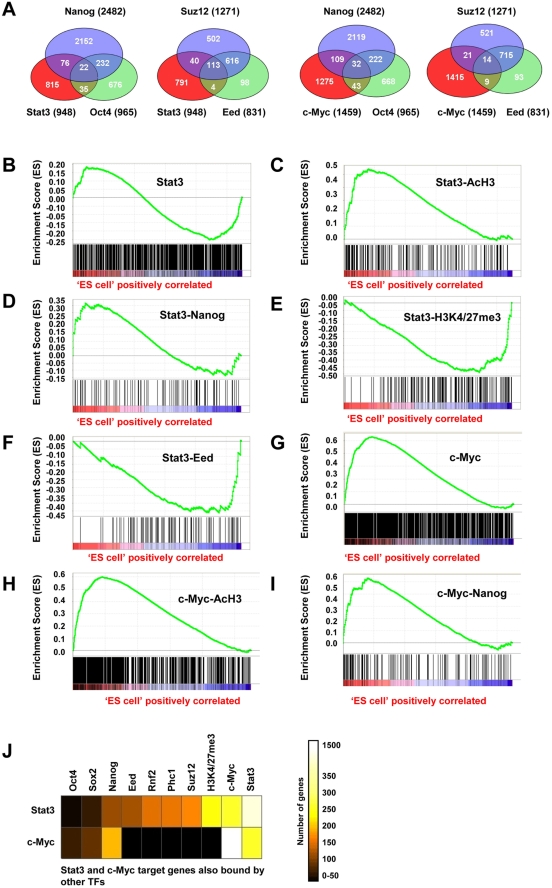
Association between TF co-occupancy and expression profile in ES cells and EBs. (A) Relationship between Stat3 and c-Myc bound genes with Nanog, Oct4, Eed, and Suz12 bound genes. GSEA analysis of (B) Stat3 target genes, (C) Stat3 target genes with AcH3 marks, (D) Stat3/Nanog target genes, (E) Stat3 target genes with H3K4/27me3 marks, and (F) Stat3/Eed target genes in ES cells and differentiated EBs. GSEA analysis of (G) c-Myc target genes, (H) c-Myc target genes with AcH3 marks, and (I) c-Myc/Nanog target genes in ES cells and differentiated EBs. Red indicates active genes in ES cells and blue indicates active genes in EBs. (J) Relationship between Stat3 and c-Myc target genes, Oct4/Sox2/Nanog target genes, polycomb repressive complex 1 (Rnf2/Phc1), polycomb repressive complex 2 (Suz12/Eed), and genes with histone marks H3K4/27me3. The number of genes co-bound by Stat3 or c-Myc is displayed.

Recent evidence suggests that genes co-bound by multiple pluripotency genes are highly expressed in ES cells, while genes bound by one or few pluripotency genes are generally inactive or are expressed at low levels [21]. Additionally, polycomb group (PcG) proteins have been shown to repress developmental genes in ES cells [29], and H3K4/27me3 bivalent chromatin configurations have been associated with developmentally repressed genes [30]. To investigate if there is a relationship between the expression state of Stat3 and c-Myc targets and co-occupancy by pluripotency factors, polycomb group proteins, and histone modifications, we compared Stat3 and c-Myc target genes with Nanog [17] and Eed [29] target genes, and genes with AcH3 (this paper) and H3K4/27me3 [30] histone marks. Stat3 targets were co-bound by Nanog at 98 genes, while c-Myc targets were co-bound by Nanog at 141 genes (Figure 6A; Table S3). Stat3 targets were co-bound by Eed at 117 genes, while c-Myc targets were co-bound by Eed at only 23 genes (Figure 6A; Table S3). Gene set enrichment analysis (GSEA) [31] was used to evaluate the expression profile of co-bound genes and genes with active and repressive histone modifications in undifferentiated ES cells and EBs [27] (Figure 6). Stat3 targets were expressed in ES cells or EBs (Figure 6B), confirming that Stat3 binds active and inactive genes in ES cells (as shown above). Actively transcribed Stat3 targets were associated with AcH3 regions in ES cells (Figure 6C), or co-occupancy with Nanog (Figure 6D), while inactive or repressed Stat3 target genes were associated with regions of H3K4/27me3 (Figure 6E), or co-binding with Eed (Figure 6F), and to a lesser extent Nanog (Figure 6D). We observed a slightly greater association between Stat3/Nanog co-bound targets and elevated ES cell gene expression compared with Stat3 target genes alone, without filtering for Nanog co-occupancy. GSEA was also used to evaluate the expression profile of c-Myc target genes in ES cells and EBs. c-Myc targets were expressed predominantly in ES cells (Figure 6G), confirming that c-Myc binds active genes in ES cells (as shown above). c-Myc targets were associated with AcH3 regions in ES cells (Figure 6H), and co-occupied with Nanog (Figure 6I), demonstrating that a subset of c-Myc target genes are co-bound by Nanog.

While Stat3 and c-Myc co-occupy a significant number of target genes (218 genes, Figure 1F) that are active in ES cells, genes bound by Stat3 without c-Myc are both active and inactive in ES cells. To evaluate the degree of co-occupancy between Stat3 and c-Myc promoter binding and pluripotency-related TFs (Oct4 [17], Sox2 [16], Nanog [17], PcG proteins [29] (Eed, Rnf2, Phc1, and Suz12), and genes with bivalent histone modifications [30] (H3K4/27me3), we compared our Stat3/c-Myc target genes with published ChIP-chip and ChIP-seq studies in ES cells. Many Stat3 targets were also bound by c-Myc, Suz12, Phc1, Rnf2, Eed, and Nanog, and contained H3K4/27me3 histone marks, while co-occupancy with Oct4 and Sox2 was found at fewer genes (Figure 6A,J). On the contrary, many c-Myc targets were co-bound by Nanog, while co-occupancy with Eed, Rnf2, Phc1, Suz12, Oct4, and Sox2, and H3K4/m27me3 histone marks was found at fewer genes (Figure 6A,J). Altogether, these results demonstrate that Stat3 and c-Myc promoter co-occupancy with pluripotency-related TFs and genes with histone H3 acetylation is associated with active target genes, while co-occupancy with polycomb repressive TFs or repressive histone modifications is associated with transcriptionally inactive target genes in ES cells.

### Stat3 targets are differentially expressed in ES cells following removal of LIF

**Figure 7:**
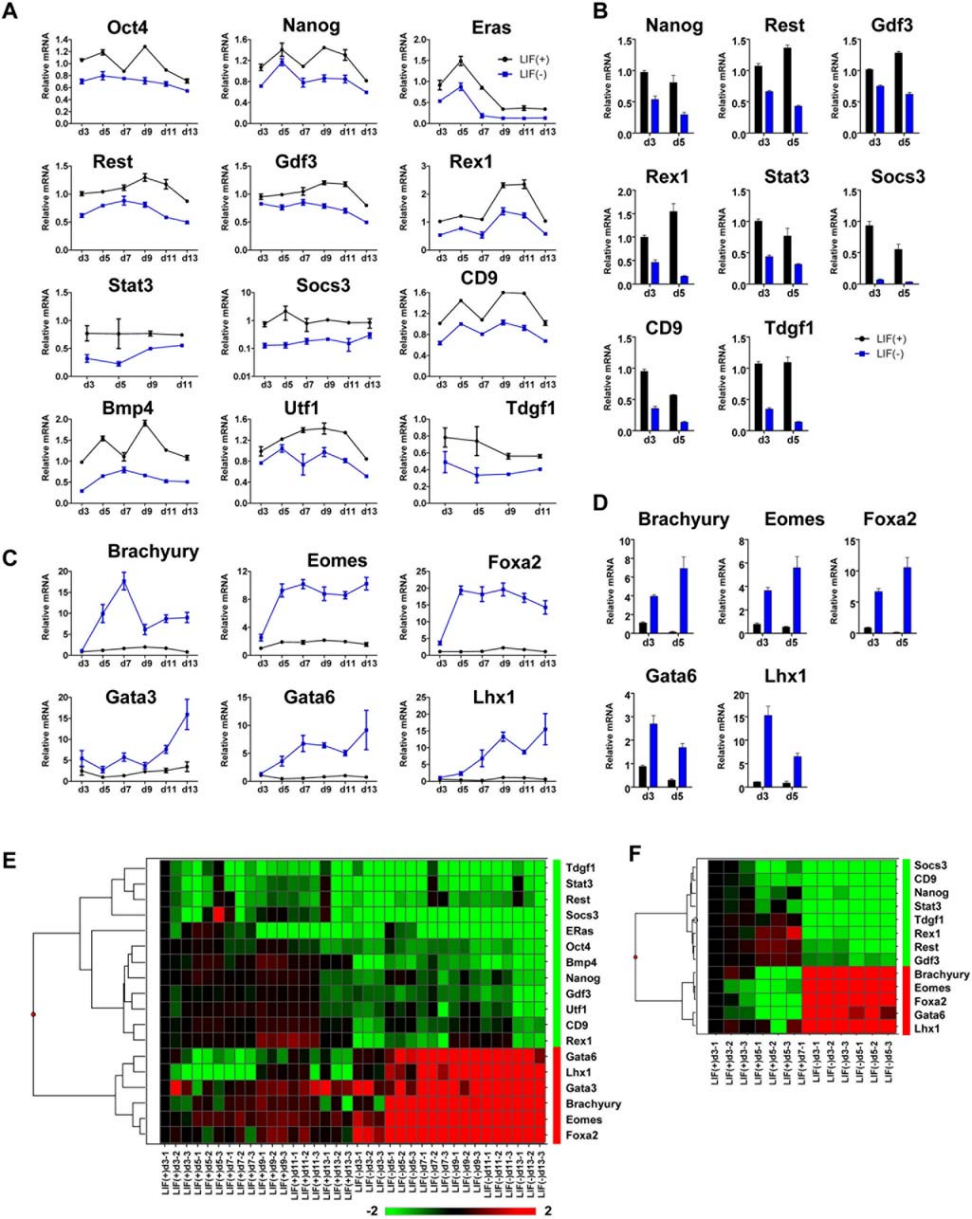
Global gene expression of Stat3 and c-Myc target genes in ES cells cultured in the absence of LIF. Pluripotency-related genes and genes bound by Stat3 and c-Myc were downregulated in ES cells cultured in the absence of LIF. (A) Q-RT-PCR expression analysis of ES cell enriched genes and genes bound by Stat3 or c-Myc (Oct4, Nanog, ERas, Rest, Gdf3, Rex1, Stat3, Socs3, CD9, Bmp4, Utf1, and Tdgf1) in ES cells cultured on iMEFs with and without LIF. (B) Q-RT-PCR expression analysis of ES cell enriched genes and genes bound by Stat3 or c-Myc in ES cells cultured in feeder-free, serum-free, and LIF-containing and LIF-independent conditions. Genes indicative of differentiation were upregulated in ES cells cultured in the absence of LIF. (C) Q-RT-PCR expression analysis of lineage specific genes bound by Stat3 (Brachyury, Eomes, Foxa2, Gata3, Gata6, and Lhx1) in ES cells cultured with and without LIF. (D) Q-RT-PCR expression analysis of lineage specific genes bound by Stat3 (Brachyury, Eomes, Foxa2, Gata6, and Lhx1) in ES cells cultured in feeder-free, serum-free, and LIF-containing and LIF-independent conditions. Data was normalized to Gapdh as an internal control and then to the expression level of ES cells cultured in the presence of LIF at 3 days. Heat map summary of global gene expression in (E) ES cells cultured in the presence or absence of LIF through 13 days and (F) ES cells cultured in feeder-free, serum-free, and LIF-containing and LIF-independent conditions for 5 days. Fold-change expression values were clustered using Spotfire.

Mouse ES cells can self-renew indefinitely in the presence of interleukin-6 family member LIF. Because Stat3 is a direct target of LIF signaling in ES cells, we examined the expression state of Stat3 ChIP-chip target genes in ES cells cultured on iMEFs in the presence or absence of LIF over a time-course of 13 days using Q-RT-PCR. After 3 days of ES cell culture in the absence of LIF, we observed downregulation of ES cell enriched genes Oct4, Nanog, Rex1, Utf1, Bmp4, and ERas and Stat3 target genes Rest, Stat3, Socs3, CD9, and Tdgf1 compared with ES cells cultured in the presence of LIF (Figure 7A,E). Downregulation of these ES cell enriched genes, including Stat3 mRNA, was sustained through 13 days of culture in the absence of LIF. We also observed upregulation of genes indicative of differentiation, also bound by Stat3, in ES cells cultured in the absence of LIF. Genes whose expression was upregulated in ES cells during culture in the absence of LIF, also bound by Stat3, include genes expressed in the three germ layers and trophectodermal cells such as Brachyury (mesoderm), Eomes (trophectoderm), Foxa2 (endoderm), Gata3 (ectoderm), Gata4 (endoderm), and Lhx1 (mesoderm) (Figure 7C,E). These results suggest that Stat3 targets are differentially expressed in the absence of LIF signaling. Because some LIF is produced endogenously by iMEFs, we evaluated the expression of a subset of Stat3 ChIP-chip targets in serum-free, feeder-free, and LIF-independent conditions over a time-course of 5 days using Q-RT-PCR. After 3 days of ES cells cultured in feeder-free and LIF-independent conditions, we observed downregulation of ES cell enriched genes Nanog, Gdf3, and Rex1 and Stat3 target genes Rest, Stat3, Socs3, CD9, and Tdgf1 compared with ES cells cultured in feeder-free and LIF-dependent conditions (Figure 7B,F). We also observed upregulation of differentiation-related genes also bound by Stat3 including Brachyury, Eomes, Foxa2, Gata6, and Lhx1 in ES cells cultured in feeder-free and LIF-independent conditions (Figure 7D,F). These results further confirm that Stat3 targets are differentially expressed in the absence of LIF.

## Discussion

**Figure 8:**
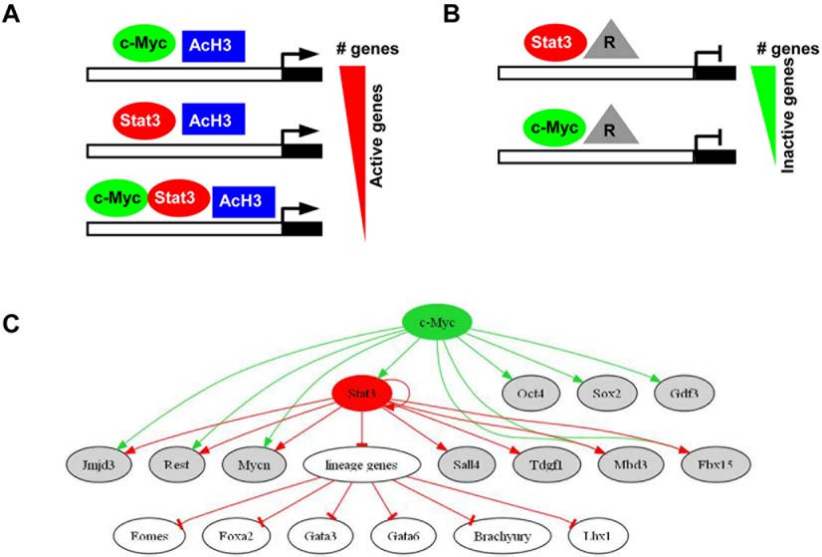
Interaction of Stat3 and c-Myc transcription factors with promoters of ES cell enriched and lineage specific genes. Transcriptional regulation of c-Myc and Stat3 target genes in ES cells. (A) Actively transcribed c-Myc and Stat3 targets are associated with regions of AcH3. c-Myc occupies a greater number of active genes than Stat3; Stat3 and c-Myc co-occupy a number of ES cell enriched genes. The red triangle represents highly expressed genes in ES cells. (B) Inactive genes occupied by Stat3 and c-Myc are bound by polycomb group proteins and are associated with regions of repressive histone modifications. The gray triangle labeled [R] represents repressive proteins and histone modifications. Stat3 occupies a greater number of inactive genes in ES cells relative to c-Myc. The green triangle represents inactivate genes in ES cells. (C) Transcriptional regulatory circuit of Stat3 and c-Myc bound genes.

Mouse ES cells have the ability to self-renew indefinitely in the presence of LIF. Although it is known that LIF signaling through Stat3 maintains ES cells in a self-renewing state, direct transcriptional targets of Stat3 have not been fully identified. However, it has been suggested that Stat3 and c-Myc share downstream targets. c-Myc can maintain ES cell self-renewal independent of LIF signaling [11], and c-Myc is also one of several proteins, along with Oct4, Sox2, and Klf4, that when co-expressed in mouse and human somatic cells, facilitates nuclear reprogramming to a pluripotent state [12]–[15]. Because Stat3 and c-Myc are necessary for establishing and maintaining a pluripotent state, evaluation of Stat3 and c-Myc downstream transcriptional targets provides further insight into the ES cell transcriptional network. Therefore, we used genome-wide chromatin immunoprecipitation and microarray analysis (ChIP-chip) to map Stat3 and c-Myc promoter binding sites in mouse ES cells. Here, we demonstrate that Stat3 and c-Myc occupy many ES cell enriched genes. Additionally, by evaluating Stat3 and c-Myc ChIP-chip target gene expression profiles in undifferentiated ES cells and differentiated EBs, we observed Stat3 binding to both active and inactive genes, while c-Myc was bound to mainly active genes in ES cells (Figure 8). Furthermore, we found that co-occupancy of Stat3 and c-Myc target genes with pluripotency-related TFs and regions of active histone modificiations is correlated with gene activity, while Stat3 co-occupancy with polycomb repressive proteins and regions of repressive histone modifications is correlated with gene inactivity (Figure 8A–C). We also observed activation or repression of Stat3 target genes in the absence of LIF signaling, concomitantly with reduced Stat3 transcript levels, suggesting that Stat3 promoter occupancy plays a role in regulating target gene expression.

Our results showing that Stat3 associates with both active and inactive genes in ES cells, while c-Myc associates with predominantly active genes, is consistent with previous reports demonstrating that Stat3 and c-Myc promote and inhibit transcription by associating with co-activators and co-repressors. Stat3 association with co-activators steroid receptor coactivator 1 (NcoA/SRC1a) and cAMP response element binding protein/p300 (CBP/p300) augment Stat3 transcriptional activity [32]. Crif1 has also been shown to be a coactivator of Stat3 [33]. Stat3 can also associate with co-repressor Kap1/Tif1β to inhibit transcription [34]–[36], where Kap1 inhibits transcription by recruiting histone deacetylase complex (Hdac) and associating with histone methyltransferase [37]–[39]. c-Myc, like Stat3, is known to associate with both co-activators and co-repressors. c-Myc can associate with co-repressor proteins Kap1/Tif1β and Hdac to negatively regulate transcription of target genes [38]. While we observed c-Myc binding to mainly active genes in ES cells, c-Myc was bound to a minimal set of inactive genes. These data suggest that Stat3 activates and represses target gene transcription in ES cells, while c-Myc mainly activates genes.

Further analysis of our ChIP-chip data showed that c-Myc occupied 135 genes encoding proteins of ribonucleotide protein complexes, and 82 genes encoding structural constituents of ribosomes. The most significant biological process overrepresented in c-Myc targets included genes involved in protein synthesis (Figure 1H). c-Myc has been shown to function in regulating gene expression of ribosomsal proteins/RNAs [40], [41]. Moreover, c-Myc induced tumorigenesis is correlated with ribosomal protein expression [42], suggesting that c-Myc induced expression of ribosomal proteins is involved in tumorigenesis. By occupying and regulating transcription of ribosomal proteins, c-Myc may promote the tumorigenic growth property of ES cells.

Pluripotency can be induced in somatic cells through cell fusion with ES cells [43], where Nanog enhances the efficiency of cell-fusion induced reprogramming [44]. Additionally, overexpression of four transcription factors (Oct4, Sox2, Klf4, and c-Myc) is sufficient to reprogram mouse embryonic fibroblasts (MEFs) [12] and human fibroblasts [13]–[15] to a pluripotent state (iPS cells). Our data shows that c-Myc binds three out of the four reprogramming factors (Oct4, Sox2, Mycn) and one other another gene, Klf5, which is functionally redundant to the reprogramming factor Klf4 [18]. While c-Myc has been shown to be dispensable for inducing pluripotency [45], the frequency of generating iPS cells is lower without Myc. c-Myc may enhance reprogramming by augmenting transcription of pluripotency inducing factors through direct promoter binding.

c-Myc has also been shown to be involved in other cellular processes such as cell cycle progression [46]. In our ChIP-chip dataset we found c-Myc promoter occupancy at many cell cycle regulatory genes including cyclin genes such as Ccnb1, Ccnd1, Ccne1, Ccnf, Ccng1, and Ccnt2; cyclin dependent kinase genes such as Cdk4, Cdk6, Cdk7, and Cdkl3; and other cell cycle regulatory genes including Cdc26, Cdc6, Cdca1, Cdca7, Cdca8, and Cdk2ap1 (Table S2). Further functional annotation of c-Myc targets using IPA identified many additional cell cycle regulatory genes bound by c-Myc such as Bard1, Bax, Ctcf, Daxx, Dnmt1, Hes1, Max, Mycn, Rb1, and Sox2. c-Myc promoter occupancy of cell cycle regulatory genes may play a role in the rapid cell division of ES cells (∼12 hrs).

In summary, genomic maps of Stat3 and c-Myc promoter binding in ES cells reveal previously unknown targets of two key genes necessary for ES cell pluripotency. Identifying targets of Stat3 and c-Myc strengthens our understanding of the ES cell transcription network. ChIP-chip studies utilizing promoter-tiling arrays are powerful in identifying protein/DNA interactions near TSSs of known genes in ES cells. However, to identify TF binding near distal enhancers or unknown genes it will be necessary to perform additional analyses such as whole-genome tiling array analysis (ChIP-chip) or sequential ChIP (ChIP-seq). Further investigation of ES cell TF networks will reveal how mechanisms of gene expression contribute to pluripotency, reprogramming, and lineage specific differentiation.

## Materials and Methods

### ES cell culture

R1 ES cells, cultured as previously described [47], were obtained from ATCC (Manassas, VA), and maintained at 37°C with 5% CO_2_ on irradiated mouse embryonic fibroblasts (iMEFs) in high-glucose containing DMEM (Gibco-BRL), 15% ES cell qualified FCS (Hyclone, Logan, UT), 1,000 units/mL LIF (Cellgro), penicillin/streptomycin, L-glutamine, non-essential amino acids, and 50 µM β-mercaptoethanol (Gibco-BRL). Feeder-free ES cells were cultured in gelatin-coated dishes and transitioned into serum-free media (ESGRO Complete, Chemicon). R1 ES cells were cultured at 37°C with 5% CO_2_ in gelatin-coated dishes in serum-free media (ESGRO complete clonal grade medium, Chemicon), passaged by washing with PBS, and dissociated with accutase (Chemicon).

### RNA isolation and Q-RT-PCR of ES cells grown with and without LIF

RNA isolation and Q-RT-PCR were performed as previously described with minor modifications [48]. ES cells were harvested using 0.25% trypsin (Invitrogen) to dissociate cells, centrifuged in 10% FCS DMEM medium to inactivate trypsin, and pelleted. To remove feeder cells, ES cells were plated in gelatin-coated dishes in ES cell media without iMEFs, and incubated at 37°C with 5% CO_2_ for 20–30 minutes. After 20–30 minutes, the medium was aspirated gently and cells were pelleted. Total RNA was extracted from ES cells cultured with or without LIF using an RNeasy Mini Kit (Qiagen, Valencia, CA) and DNase treated using Turbo DNA-free (Ambion) for 30 minutes at 37°C. Reverse transcription was performed using a Superscript III kit with random hexamer primers (Invitrogen, Carlsbad, CA). Q-RT-PCR was performed using TaqMan assays with TaqMan Universal PCR Master Mix reagents or SYBR Green PCR Master Mix reagents (Applied Biosystems).

### Chromatin immunoprecipitation and DNA microarray analysis (ChIP-chip)

The polyclonal Stat3 antibody (C-20, SC-482) and the polyclonal c-Myc antibody (N-262, SC-764) were obtained from Santa Cruz Biotechnology. The polyclonal AcH3 (acetyl-histone-H3, #06-599) and rabbit IgG (PP64B) antibody were obtained from Upstate. 1×10^8^ mouse R1 ES cells (feeder-free) were harvested and chemically crosslinked with 1% formaldehyde (Sigma) for 20 hours at 4°C. Fixation was quenched by addition of 1/20 volume 2.5 M glycine for 5 minutes at room temperature. Cells were pelleted at 4°C (500 g), washed with ice-cold 1× PBS, washed twice with lysis buffer (10 mM Tris-HCL, 10 mM NaCl, 3 mM MgCl_2_, 0.5% GEPAL, 1 mM fresh PMSF), pelleted, and flash frozen in liquid nitrogen. Pellets were resuspended in pre-IP dilution buffer (10 mM Tris-HCL, 10 mM NaCl, 3 mM MgCl_2_, 1 mM CaCl_2_, 4% GEPAL, 1 mM PMSF), 60 µL PMSF, and additional components (100 mM PMSF, 25× protease inhibitor, 20% SDS, 5 M NaCl, H_2_O). Cells were sonicated using a Branson Sonifier 450D at 50% amplitude, with 12×1 minutes pulses, followed by 1 minute rests in ice water. Sonicated fragments ranged in size from 200–1000 bp. Post sonication, samples were centrifuged at 14,000 rpm for 10 minutes at 4°C, aliquoted, and flash frozen in liquid nitrogen. Sonicated cell extracts equivalent to 2×10^6^ cells were used in subsequent immunoprecipitations. Samples were pre-cleared with protein G Dynabeads (Dynal) in 1000 µL dilution buffer (0.01% SDS, 1.1% Triton X-100, 1.2 mM EDTA, 16.7 mM Tris-HCl (pH 8.1), 167 mM NaCl, 5 µL Upstate protease inhibitor cocktail II). Cell extracts were incubated with 1 µg antibody overnight at 4°C. Chromatin-antibody complexes were isolated with 100 µL protein G Dynabeads, and washed one time with low salt buffer (0.1% SDS, 1% Triton X-100, 2 mM EDTA, 20 mM Tris-HCl, 150 mM NaCl), one time with high salt buffer (same as low salt with 500 mM NaCl), one time with LiCl wash buffer (0.25 M LiCl, 1% IGEPAL-CA630, 1% deoxycholic acid, 1 mM EDTA, 10 mM Tris), and twice with TE. Protein/DNA complexes were eluted from the beads in 10 µL 20% SDS, 20 µL 1 M NaHCO_3_, and 170 µL H_2_O at 65°C with occasional vortexing. Crosslinking was reversed by addition of 8 µL 5 M NaCl and incubation overnight at 65°C. Extracts were then treated with RNase A and proteinase K, and DNA was purified using an Upstate EZ ChIP kit. 5 µg purified DNA (Stat3-chip, c-Myc-chip, AcH3-chip, and Input) was amplified using a GenomePlex® Whole Genome Amplification (WGA) Kit (Sigma), DNase treated, and labeled with a GeneChip® WT Double-Stranded DNA Terminal Labeling Kit. Labeled DNA was hybridized to Affymetrix mouse promoter 1.0R tiling arrays for 16 hours at 45°C in an Affymetrix GeneChip® Hybridization Oven 640, washed using a GeneChip® Fluidics Station 450, and scanned with a GeneChip® Scanner 7G at EMD SRI. Immunoprecipitated (Stat3, c-Myc, and AcH3) and non-immune (rIgG) control sample biological triplicates were used for ChIP-chip analysis. Mouse promoter 1.0R DNA tiling arrays contain 4.6 million 25-mer oligonucleotide probes covering a distance of -6kb to +2.5kb relative to the transcriptional start site for 28,000 mouse promoter regions annotated from ENSEMBL genes, RefSeq mRNAs (NCBI GenBank®), and complete-CDS mRNAs (NCBI GenBank®). These tiling arrays provide a resolution of 35 bp with 10 bp gaps between probes.

### Peak detection and gene annotation

Quantile normalization, including probe intensity computation and log2 adjustment was applied to Affymetrix tiling array data (Stat3, c-Myc, and Input) using CisGenome (http://www.biostat.jhsph.edu/~hji/cisgenome/). Peak detection was done using the TileMap [22] (http://biogibbs.stanford.edu/~jihk/TileMap/index.htm) application in CisGenome. MA statistics was applied to analyze the tiling array data [22]. Post filtering included discarding peaks if the total length was less than 100 bp or there were less than 3 continuous probes passing the cutoff. Peak post filtering also included merging two adjacent peaks if the gap between the two peaks was less than 300 bp and the there were less than 5 probes that did not pass the cutoff between the two peaks. FDR statistics (left tail) were also estimated. TileMap utilizes a two-step approach for analyzing DNA binding regions. First, a hierarchical empirical Bayes model is used to compute a test-statistic for each probe [22]. Second, probe test-statistics in a genomic region are used to determine significance of hybridization. Enrichment peaks were annotated with the closest gene and defined by the distance upstream to the TSS and the distance downstream of the TES. Build 36 of the mouse genome was used in these analyses.

### Confirmation of ChIP-chip binding regions using Q-PCR

Primers were designed for Stat3 and c-Myc ES cell ChIP-enriched genomic DNA regions using Primer 3 (http://frodo.wi.mit.edu/). Real-time Q-PCR was performed on non-amplified Stat3, c-Myc, rabbit IgG, and Input ES cell ChIP DNA using SYBR Green Master Mix reagents with an ABI PRISM 7900HT sequence detection system.

### Microarray analysis

Annotated Stat3 and c-Myc ChIP-chip regions were compared with a published microarray dataset to evaluate developmental expression patterns of Stat3 and c-Myc bound genes. Affymetrix expression data from ES cells and embryoid bodies (EB) (GSE2972) [27] was download from Gene Expression Omni (GEO). CEL files were imported into ArrayAssist (Stratagene, Lo Jolla, CA) and probe levels were normalized using the GeneChip® Robust Multiarray Average (GCRMA) algorithm. Analysis of variance (ANOVA) was performed on all groups using a Benjamini and Hochberg false-discovery rate (FDR) correction. Hierarchical clustering analysis was performed on log-transformed statistically significant (P-value less than 5%) mean-centered data using Spotfire (Cambridge, MA).

### Supplemental Data

Supplemental data include three tables.

## Supporting Information

Table S1 Stat3 bound regions in murine ES cells
(0.11 MB XLS)

Table S2 c-Myc bound regions in murine ES cells
(0.17 MB XLS)

Table S3 Stat3 and c-Myc occupied genes co-bound with Oct4 and Nanog
(0.15 MB XLS)
